# How smart is artificial intelligence in organs delineation? Testing a CE and FDA-approved Deep-Learning tool using multiple expert contours delineated on planning CT images

**DOI:** 10.3389/fonc.2023.1089807

**Published:** 2023-03-02

**Authors:** Silvia Strolin, Miriam Santoro, Giulia Paolani, Ilario Ammendolia, Alessandra Arcelli, Anna Benini, Silvia Bisello, Raffaele Cardano, Letizia Cavallini, Elisa Deraco, Costanza Maria Donati, Erika Galietta, Andrea Galuppi, Alessandra Guido, Martina Ferioli, Viola Laghi, Federica Medici, Maria Ntreta, Natalya Razganiayeva, Giambattista Siepe, Giorgio Tolento, Daria Vallerossa, Alice Zamagni, Alessio Giuseppe Morganti, Lidia Strigari

**Affiliations:** ^1^Department of Medical Physics, IRCCS Azienda Ospedaliero-Universitaria di Bologna, Bologna, Italy; ^2^Medical Physics Specialization School, Alma Mater Studiorum, University of Bologna, Bologna, Italy; ^3^Department of Radiation Oncology, IRCCS Azienda Ospedaliero-Universitaria di Bologna, Bologna, Italy; ^4^Department of Medical and Surgical Sciences (DIMEC), Alma Mater Studiorum, University of Bologna, Bologna, Italy

**Keywords:** deep learning tool, segmentation, independent external validation, quality metrics, time saved

## Abstract

**Background:**

A CE- and FDA-approved cloud-based Deep learning (DL)-tool for automatic organs at risk (OARs) and clinical target volumes segmentation on computer tomography images is available. Before its implementation in the clinical practice, an independent external validation was conducted.

**Methods:**

At least a senior and two in training Radiation Oncologists (ROs) manually contoured the volumes of interest (VOIs) for 6 tumoral sites. The auto-segmented contours were retrieved from the DL-tool and, if needed, manually corrected by ROs. The level of ROs satisfaction and the duration of contouring were registered. Relative volume differences, similarity indices, satisfactory grades, and time saved were analyzed using a semi-automatic tool.

**Results:**

Seven thousand seven hundred sixty-five VOIs were delineated on the CT images of 111 representative patients. The median (range) time for manual VOIs delineation, DL-based segmentation, and subsequent manual corrections were 25.0 (8.0-115.0), 2.3 (1.2-8) and 10.0 minutes (0.3-46.3), respectively. The overall time for VOIs retrieving and modification was statistically significantly lower than for manual contouring (p<0.001). The DL-tool was generally appreciated by ROs, with 44% of vote 4 (well done) and 43% of vote 5 (very well done), correlated with the saved time (p<0.001). The relative volume differences and similarity indexes suggested a better inter-agreement of manually adjusted DL-based VOIs than manually segmented ones.

**Conclusions:**

The application of the DL-tool resulted satisfactory, especially in complex delineation cases, improving the ROs inter-agreement of delineated VOIs and saving time.

## 1 Introduction

Automation-based solutions are spreading in several medical sectors, including radiotherapy (RT), finding applications in the entire workflow (1).

Advanced techniques for optimal RT require the careful delineation of the target and organ at risk (OARs) to obtain an accurate and precise dose distribution for fixed and moving targets. Unfortunately, one of the critical issues remains the accuracy and reproducibility of target and OARs segmentation on Computed Tomography (CT) images in the treatment planning stage. Although contouring guidelines of the OARs for the different anatomical districts have been proposed (European Society for Therapeutic Radiology and Oncology (ESTRO) and Radiation Therapy Oncology Group (RTOG) guidelines) (2–12), significant inter-/intra- user variability has been reported (13, 14), mainly correlating with prior knowledge and experience of radiation oncologists (ROs). Such inconsistencies from manual contouring can affect the evaluation of effective doses delivered to the OARs, maximizing toxicity or biasing target coverage. In addition, manual segmentation might be subject to human error and requires the application of robust checklists and guidelines.

One of the main applications of artificial intelligence (AI)-based tools to RT-based patient workflow is organ and target segmentation. In this context, different commercial systems developed AI-based auto-segmentation tools such as MVision AI Oy (Helsinki, Finland) (15), Limbus (Limbus AI Inc, Regina, SK, Canada) (16), MIM Contour Protégé AI™ (MIM Software Inc., Cleveland, OH) (17), promising support, standardization and shortening the time of manual segmentation. However, the number of cases used to assess AI tool-added value and accuracy in RT departments is limited to a few tumor sites, a few patients, and a few VOIs.

MVision AI Oy is a CE- and FDA-approved cloud-based Deep Learning (DL) software for automatic OAR segmentation of CT images, trained and tested on VOIs delineated according to the ESTRO and RTOG guidelines in training and test centers. To date, organ auto-segmentation efforts have been mainly focused on adult populations using a unique manual segmentation as the gold standard. However, multiple manual segmentations per structure coming from different medical experts are recommended to produce high-quality results (18). The variability in contouring among ROs is a crucial issue because manual contouring still represents one of the significant causes of uncertainty in the RT workflow, although ROs follow the same international guidelines. So, when a system trained and tested in some centers is applied in a new center, proposing to the ROs different contours from those manually made in the new center.

This study aims to present the developed frameworks and the platform for assessing the agreement of the unmodified automatic VOIs obtained from MVision, the manual/semi-automatic VOIs, and the modified MVision VOIs performed by experienced or in-training ROs. Based on expert contours of ROs with different levels of experience and trained in multiple centers, we measured inter-ROs variability of manual contours and estimated the agreement or disagreement of DL-tool-based contours accordingly to the district and VOIs. In addition, we estimated the inter-ROs variability of DL-tool-based contours manually adjusted. Moreover, we investigated the impact of the type of scanner, acquisition and reconstruction parameters, presence of contrast medium, and the potential reasons for which the DL-tool correctly did not perform the contour or failed to identify a VOI. In addition, we studied the degree of satisfaction with the tool from poor to good, and the possible causes of contours rejected or requiring substantial modifications.

For the above reasons, this study represents an additional independent external validation of this DL-based algorithm before its application in our RT department, which already follows international guidelines for contouring. To our knowledge, a comprehensive platform for assessing the accuracy and reproducibility of introducing a new AI tool for automatic contouring in different anatomical districts is still missing.

## 2 Materials and methods

### 2.1 Patients & investigated tumor sites

To investigate the performance of MVision, we selected six anatomical districts with VOIs of different sizes, shapes, and electron densities and its robustness regarding the CT acquisition and reconstruction parameters adopted in our center. A minimum of 20 patients per district were selected except for female pelvis to generate a dataset representative of patients treated in our Institute. We aimed to cover most of the districts treated in our everyday clinical practice.

CT images were acquired with CT Brilliance Big Bore (Koninklijke Philips Electronics NV, Amsterdam, NL) or Discovery STE (GE Healthcare, Chicago, Illinois, USA). The native slice thickness of the acquired images was 3.75 mm for the GE scanner (10% of the cases), while it was variable for the Philips scanner, resulting in 2 mm (3% of the cases), 3 mm (60% of the cases), 5 mm (27% of the cases), according to our institutional disease-specific clinical protocols. In addition, the contrast medium was used in 20 patients (i.e., 18% of the investigated cohort) belonging to three districts (i.e., abdomen, H&N, and thorax). In details, 15 subjects received a CT with intravenous contrast medium to better define vascular structures and one received a CT with oral contrast medium to better visualize the gastrointestinal tract. In four cases both contrast media were administered during the imaging.

One-hundred-eleven patients were extracted from our Record & Verify (R&V) system (Mosaiq, Elekta Medical Systems), transferred, and imported to Pinnacle v.16.2 (Philips Medical Systems^®^, 61 Fitchburg, WI) treatment planning system (TPS).

The dataset represents random cancer patient cases undergoing RT according to standard clinical indications in our department. The investigated patients’ age ranged from 32 to 87 years, with a median age of 65 years. The cohort included 61 males and 50 females extracted from the database of patients treated in IRCCS Azienda Ospedaliero-Universitaria di Bologna institute and included in sub-studies approved for each district by the Ethics Committee of IRCCS Azienda Ospedaliero-Universitaria di Bologna (Identification codes of the projects: ICAROS 311/2019/Oss/AOUBo, ES-THER 973/2020/Oss/AOUBo, PORTO 533/2021/Oss/AOUBo, PAULA 201/2015/O/Oss, BREATH 229/2019/Oss/AOUBo). **Table 1** shows the number of patients for each anatomical district, tumor type, and contoured OARs with the corresponding name.

**Table 1:** Number of patients and delineated OARs for each investigated anatomical district.

#Pts [M/F]	Anatomical district	#OARs	OARs name	RO & experience	# of analyzed VOIs
20 [15/5]	Head & Neck	15	Bone_Mandible, Brainstem, Cavity_Oral, Esophagus_S, Eye_L, Eye_R, Glnd_Thyroid, Glottis, Lens_L, Lens_R, OpticNrv_L, OpticNrv_R, Parotid_L, Parotid_R, SpinalCanal	1 S 2 J	2100
20 [0/20]	Breast	14	Breast_R, Breast_L, Heart, LN_L1, LN_L2, LN_L3, LN_L4, LN_IMN, LN_Intpect, Lung_L, Lung_R, SpinalCanal, Trachea	1 S 2 J	1960
21 [13/8]	Abdomen	5	Kidney_L, Kidney_R, Liver, SpinalCanal, Stomach	2 S 2 J	945
20 [13/7]	Thorax	6	Esophagus, Heart, Lung_L, Lung_R, SpinalCanal, Trachea	2 S 2 J	1080
20 [20/0]	Male pelvis	8	Bag_Bowel, Bladder, Femur_L, Femur_R, PenileBulb, RectoSigmoid, Rectum, SeminalVes	1 S 2 J	1120
10 [0/10]	Female pelvis	8	Bag_Bowel, Bladder, Femur_L, Femur_R, Kidney_L, Kidney_R, Rectum, SpinalCanal	1 S 2 J	560
111 [61/50]	Total	59			7765

The OARs names are self-explaining. The letters L and R indicate left and right, instead LN stands for lymph node. J and S indicate junior and senior radiation oncologists, respectively.

### 2.2 Study design

The study was based on patients’ CT images from the six human body districts most frequently treated in our Institute (i.e., head-neck, breast, abdomen, thorax, male and female pelvis).

CT images were randomly extracted from our clinical database to represent the patients treated in each investigated district. In particular, our study focused on the delineation of OARs, clinical target volumes (CTVs) (i.e., breast and seminal vesicles), and elective lymph nodes (LNs) (i.e., loco-regional LNs for breast). Window/levels were selected by each physician based on the contouring consensus guidelines of the specific district and according to the RO expertise. All the contours were delineated on axial slices.

The Volumes of interest (VOIs) were delineated by senior (S=senior) and in-training (J=junior) radiation oncologists (ROs) according to our institutional protocols based on ESTRO and RTOG guidelines. S and J indicated ROs with at least ten years and at least three months of experience in cancer or pathologies of a specific district, respectively.

Contours were grouped according to the experience of ROs for each analyzed tumor site. At least one S and two Js manually contoured the investigated OARs summarized in **Table 1** using the Pinnacle TPS. The VOIs of the OARs manually contoured were identified as manually delineated (MD). Atlas-based approach available in Pinnacle was permitted only for lung segmentations as in clinical practice.

Subsequently, CT images were sent to the MVision cloud-based software (algorithm version 1.2.1 and research version for LNs delineation) for the automatic OAR segmentation and reimported in the Pinnacle workstation. Of note, for breast and prostate cancer, MVision delineates the breast, the prostate, and LNs, included in the OARs delineation analysis (See **Table 1**). All the OARs and targets available in the versions mentioned above of the software were used for the subsequent VOI comparisons. Details about MVision model architecture and libraries are reported by Olsson et al. (19).

**Figure 1:**
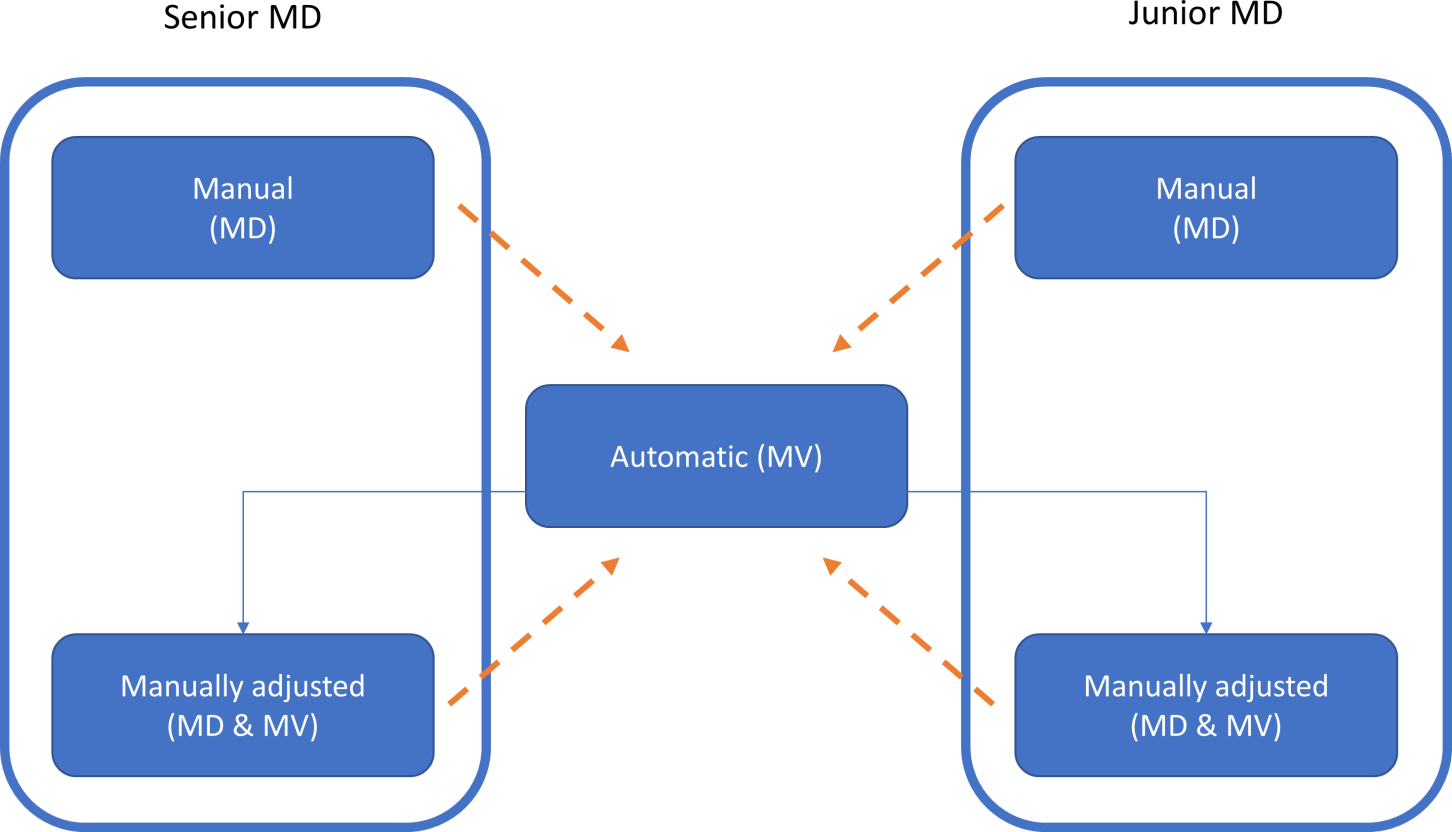
The workflow of this study for the generation of the VOIs is illustrated by using the box and solid arrows. MD, MV, and MD & MV indicate manual, automatic, and manual adjustment of the automatic delineated VOIs, respectively. The dashed arrows indicate the data comparison versus the MV VOIs for all the investigated metric indexes. A similar comparison was performed using the relative volume (rV) of manually and manually adjusted MV VOIs versus the MV ones.

The VOIs automatically segmented by MVision (in the following indicated as MV) were sent to MIM. A copy of MV VOIs was also manually adjusted by all the involved operators (named MD&MV), if necessary, as illustrated in **Figure 1**. The time between manual and MV-modified VOIs segmentation was at least six months to reduce potential bias.

For each patient and contour session, we also registered the contouring task’s duration (in minutes) and a satisfactory grade according to the Likert scale related to the automatic contour. The satisfactory grade ranged from poor (1) to excellent (5). More in detail, the adopted scale corresponded to MVision VOIs rejected and needing complete re-contouring by ROs (1), or needing major (2), some (3), minor editing (4), or not needing editing (5).

### 2.3 Data extraction and analysis

All the sets of DICOM RT structures and CT images were transferred to the MIM software (MIM Software Inc., Cleveland, OH) for the subsequent data export and analysis.

The “Statistics” module of the MIM software was used to extract the volumes (in mL) of the delineated VOIs for each district, patient, segmentation strategy, and physician code (e.g., S1 identifying the S-RO #1). The volumes were compared using the relative volume (rV) measure described in paragraph 2.3.1.

For each VOI, the “Compare Contours” module in the MIM software was used to compare the segmentation strategies (e.g., MD or MD&MV) and the physician experience (S or J) versus MV using similarity metrics. Specifically, the DICE Similarity Coefficient (DSC) and the Mean Distance to Agreement (MDA) were calculated to compare volumetric regions and voxel-wise distances, respectively. The definition of these indexes is reported in paragraph 2.3.2.

#### 2.3.1 Relative volume

The rV index is the ratio of investigated volume *V_X_* (i.e., MD or MD&MV) compared to one obtained using MVision (*V_MV_*), and it is determined as:


$$rV=\frac{V_{X}}{V_{MV}}$$

This index (expressed in an arbitrary unit, i.e., a.u.) is a metric equivalent to the relative volume difference (i.e., *RVD*) defined by Ahn et al. (20) and is:


rV=RVD+1

This index allows for assessing the organ volume variability among delineated VOIs. A good agreement among VOIs was assumed when the rV was between the same percentage of agreement observed among senior ROs.

#### 2.3.2 Similarity metrics

The DSC describes the similarity of two regions by relating the overlapping volume and the volumetric average of the volumes of interest (21), indicated as volumes V1 and V2 by:


$$DSC\left(V_{1},V_{2}\right)=\frac{2\left|V_{1}\cap^{\;}V_{2}\right|}{V_{1}+V_{2}}$$

A DSC of zero indicates that the two volumes do not overlap; instead, a DSC of one indicates that the volumes V1 and V2 are identical.

The DSC values were used as a metric for the evaluation of the DL-tool performance. Specifically, the impact on DSC values of CT slice thickness and scanner type, dependent on the acquisition and reconstruction parameters clinically adopted in our Institute, was assessed.

MDA describes the mean voxel-wise comparison of the distance between two associative points in the contour sets A and B (22), defined by:


$$MDA\left(A,B\right)=mean_{a}\in_{A,b}\in_{B}(d(a,B)\cup d(b,A))$$

MDA denotes a measure of average similarity between two contour sets with a MDA=0 indicating that sets A and B are identical.

#### 2.3.3 Data analysis

To consider the RO variability of VOI delineation, we calculated the median [range] of similarity indexes among groups for each investigated district and OAR (data not shown). For all the VOIs with DSC<0.5 between MV and MD or MD&MV, a board-certified RO analyzed in blind the VOI adherence to ESTRO guidelines and registered the potential causes of the discrepancies.

The similarity indices, the satisfactory grade of using the DL-tool, and time saved were shown using boxplots, Pyramid plots, or tables. The level of satisfaction and the saved time was analyzed using the Spearman test. Receiver Operating Characteristic (ROC) curve analysis was conducted to investigate the predictors of a satisfactory score≥4, (i.e., DSC and MDA). The significance among groups was assessed by using Wilcoxon or t-test when comparing two groups, as appropriate, while using ANOVA or Tuckey in case of more than two groups. A p-value<0.05 indicated the statistical significance between groups.

We developed all the data analysis using an *ad hoc* R tool, described in more detail in paragraph 2.3.2, on available datasets generated using MIM software.

#### 2.3.4 Description of R tool for data analysis

We created an R tool that allows the automatic import of the.csv files related to volumes and similarity comparison, respectively, for each investigated district.

The possible different VOI names were harmonized, and the data were reorganized in a database. The VOIs with different laterality (e.g., breast, lung, etc.) were aggregated in a single OAR except for breast lymph nodes.

For each district, we calculated the occurrences of DSC and MDA values overcoming a threshold as defined in (13) in MD and MD&MV, respectively. Specifically, we used a threshold of 0.8 and 3 mm for DSC and MDA values, respectively. The results were shown using Pyramid plots, where the height of the bar is proportional to the number of cases in each group. Moreover, the boxplots of volumes among groups for the most representative differences highlighted using Pyramid plots were shown.

For each investigated expertise, district, and VOI, we performed a boxplot of MV, MD, MD&MV volumes (cc), and rV(a.u.). The ANOVA one-way test was performed to assess the overall statistical significance between groups, and the two-way t-test was used to compare the MD and MD&MV volumes against MV ones, taken as the reference.

## 3 Results

### 3.1 Delineation time, quality of auto-contouring, and segmented VOIs

Seven thousand sixty-five (7765) VOIs were delineated on the CT images of 111 patients, representative of 6 tumor sites. The median (range) time for manual contouring was 25.0 minutes (8.0-115.0), for retrieving auto-segmented VOIs was 2.3 minutes (1.2-8.0), while for the subsequent manual corrections was 10.0 minutes (0.3-46.3). The overall time for retrieving VOIs and their modification was 12.3 minutes (1.9-48.8), resulting in statistically significantly lower than that of the manual contouring (p<0.001). The median (range) time for manual contouring in S and J subgroups was 25.0 minutes (11.1-57.9) and 24.0 minutes (8.0-115.0), respectively, while the total time for retrieving auto-segmented VOIs and performing the subsequent manual corrections was 12.0 minutes (1.9-25.3) and 13.3 minutes (2.1-48.8), respectively. For both S and J subgroups, the time required for obtaining and eventually manually adjusting the VOIs from the DL-tool was statistically significant to the time employed for the manual contouring alone (p<0.001), thus not depending on the MDs’ experience.

By considering each investigated district separately, the median (range) difference time between manual contouring compared with manual adjustment after the retrieving of auto-segmented VOIs was 21.5 minutes (0.4-52.7) for the abdomen, 11.2 minutes (6.7-17.9) for the thorax, 9.5 minutes (4.5-66.3) for the H&N, 8.7 minutes (-0.3-25.7) for the breast, 6.9 minutes (-3-16.0) for the female pelvis, and 13.7 minutes (3.5-18.4) for the male pelvis. The median percentage of time saved was 69%, 79%, 67%, 48%, 29%, and 53% for the abdomen, thorax, H&N, breast, female and male pelvis, respectively, while the maximum percentage time saved was 92%, 89%, 63%, 73%, 54%, 72% for abdomen, thorax, H&N, breast, female and male pelvis, respectively. Only in three cases (one breast and two female pelvis) the time difference of a single RO was negative, mainly due to the prolonged time employed for the manual adjustment after the auto-segmented VOIs retrieval.

Overall, the system was generally appreciated by ROs, with 44% of vote 4 (well done) and 43% of vote 5 (very well done) correlated with the spared time for the delineation correction after MV automatic contouring (p<0.001).

**Figure 2:**
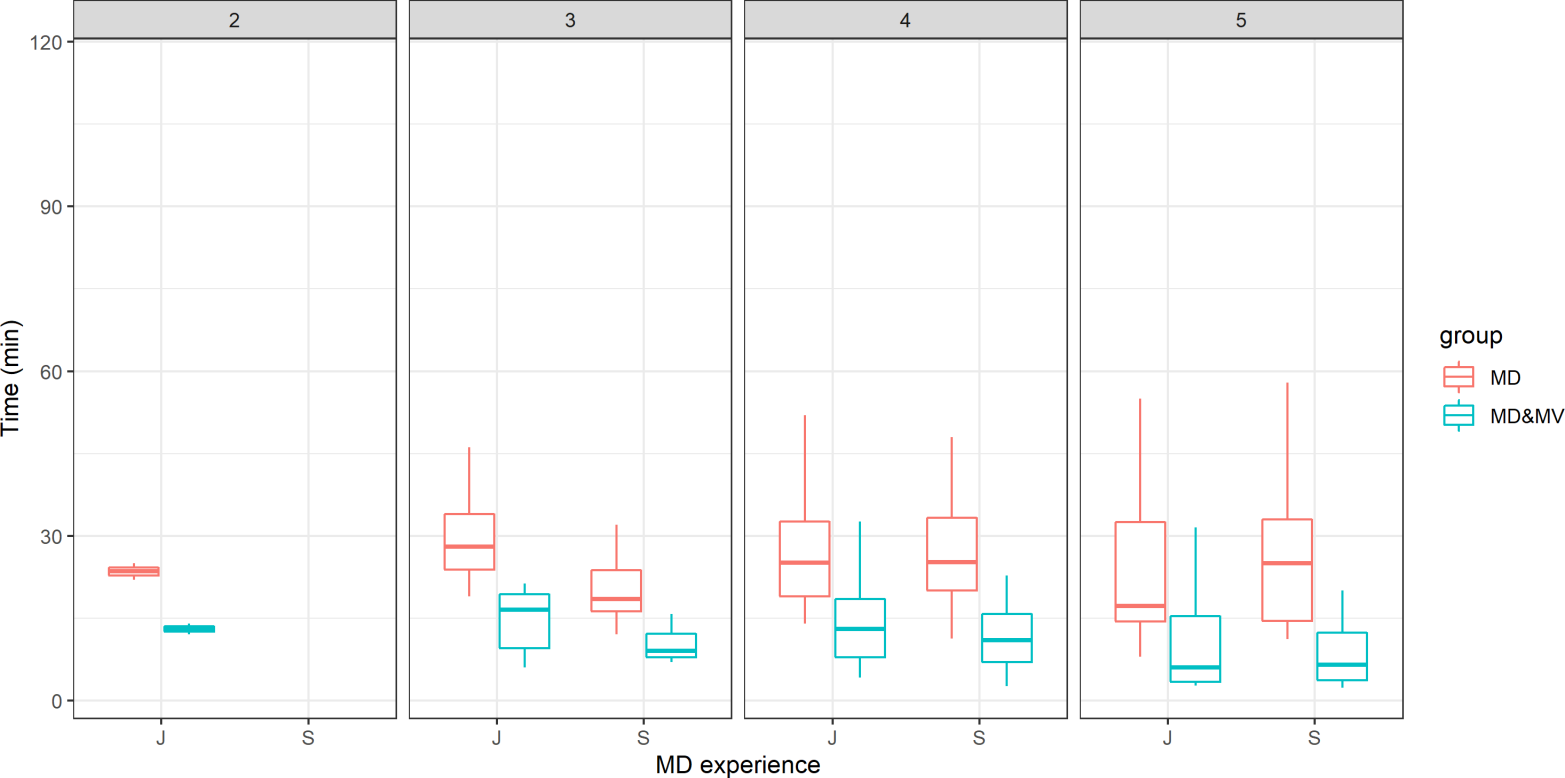
The time for the VOI delineation in minutes versus the ROs' experience (J=junior vs S=senior) and the score representing the grade of satisfaction of the DL tool-based auto-contouring ranging from 1 (poor) to 5 (excellent).

The time for the contouring was similar in terms of manual delineation, irrespective of ROs’ experience or satisfactory grade with the tool (**Figure 2**). Moreover, the time of manual adjustment was statistically significantly lower in all the above scenarios (ROs’ experience or satisfactory grade). The satisfactory grade increases according to the time saved and indirectly according to the complexity of patients’ volume delineations. The average score was 4.08, 4.23, 4.45, 4.86, 3.7, and 4.79 for the abdomen, H&N, breast, thorax, female and male pelvis, respectively. This score was statistically significantly higher in S than in J ROs (p-value=0.01).

The area under the ROC curve predicting a satisfactory grade ≥4 using the mean DSC per patient was 0.708(95% CI: 0.509-0.907), 0.665(95% CI: 0.535-0.794), and 0.673(95% CI: 0.565-0.782) for S, J, and the whole group, respectively, while it was not statistically significant using the mean MDA per patient.

MV generally delineated all the planned VOIs reported in **Table 1** with few exceptions: a whole maxilla, two penile bulbs, four seminal vesicles, one femoral head, and three lenses.

**Figure 3:**
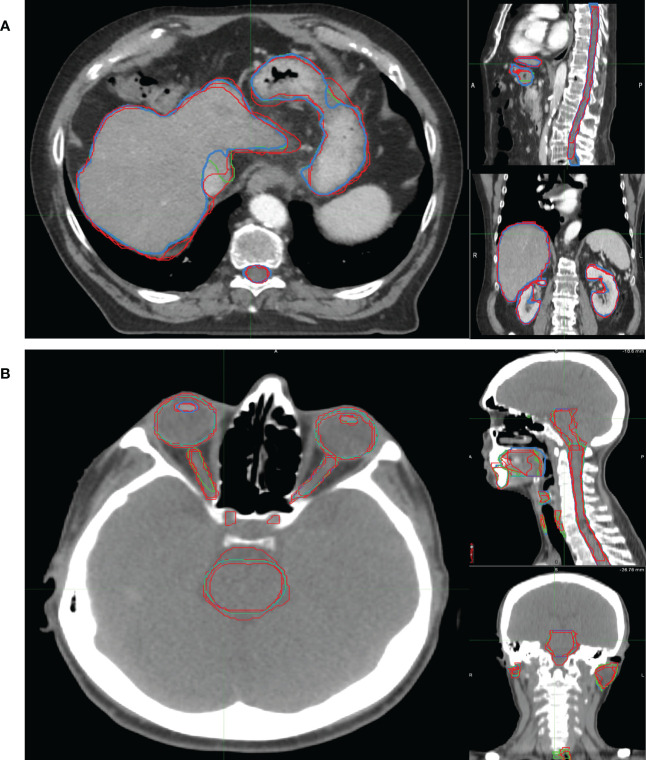
Examples of OARs delineated in axial (left), sagittal (right up) and coronal (right down) view of illustrative **(A)** abdominal and **(B)** H&N cancer patient. The blue, red and green lines rep-resent the OARs delineated by MV, MD and MD & MV, respectively. See the definition of **Figure 1** legend.

Examples of OARs delineated in two representative patients are shown in **Figure 3**.

### 3.2 Volumes

The volumes were quite similar in larger organs, with a better agreement between MD&MV versus MV than in MD versus MV (data not shown). The median agreement between VOIs among senior ROs was lower than 10%, with few exceptions (e.g., spinal canal, as explained in the following). Thus, we considered a good agreement among VOIs when rV was between 0.9 and 1.1. The differences in volumes delineated in both junior and senior RO groups were statistically significant in the MD group but not in the MD&MV group; thus, the modified MV contour volumes resulted independent of the level of ROs experience.

**Figure 4:**
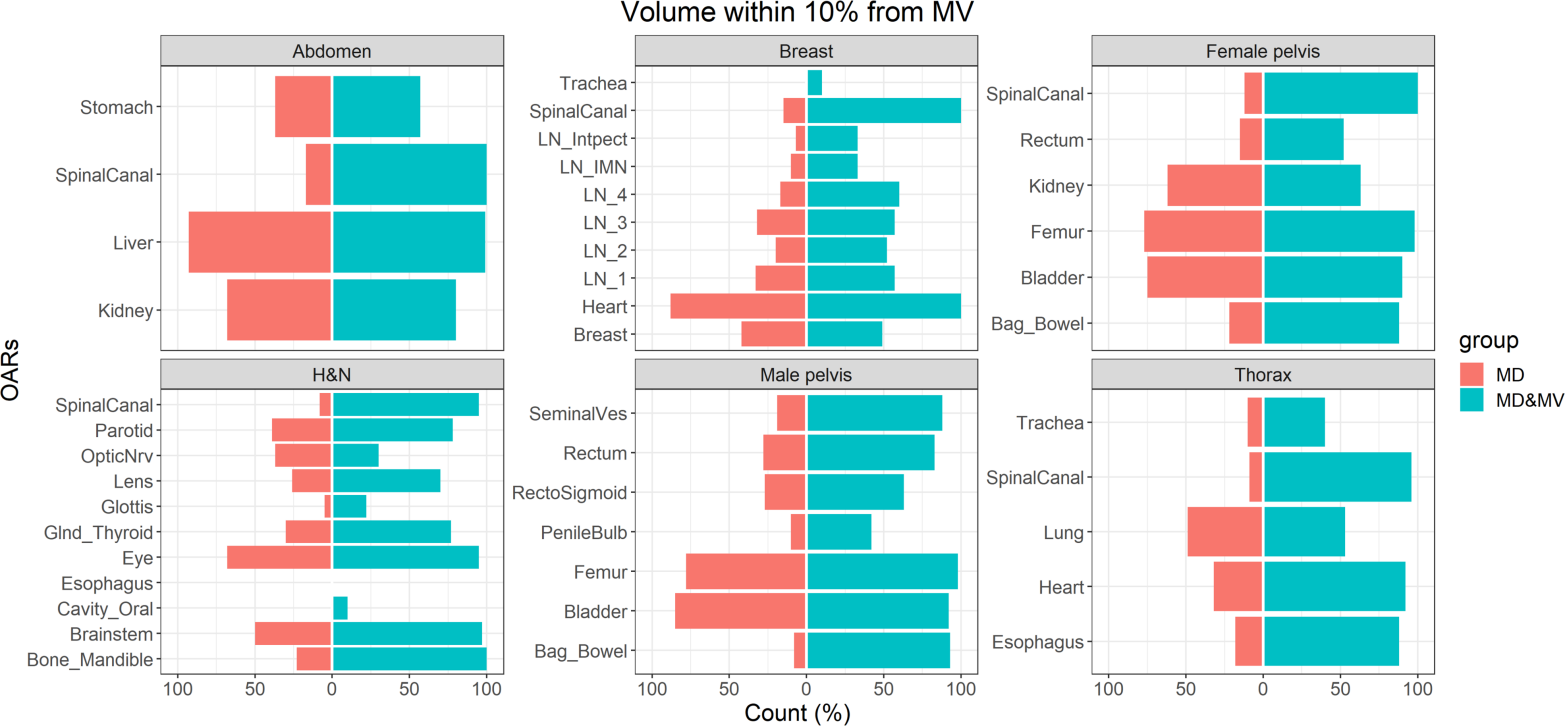
The percentage number of OARs with rV within 10% with the VOIs delineated by MV grouped for the tumoral district and type of delineation (i.e., MD or MD & MV). The absence of the colored bar (e.g., for the esophagus in the H&N district) indicates that the comparison did not produce any count as rV between each considered VOI (i.e., for each RO and type of delineation) compared to the MV-based one exceeded the 10%. See the definition of **Figure 1** legend.

**Figure 4** shows the percentage number of VOIs delineated by the MD or MD&MV group with rV between 0.9 and 1.1 compared to MV ones. The number of OARs with rV within 10% (compared to the VOIs delineated by MV) significantly increased after the manual modification of MV VOIs for most organs (**Figure 4**). Of note, all the OARs are shown except for the esophagus in the H&N district, in which both the rVs of MD and MD&MV groups exceeded 10%.

**Figure 5:**
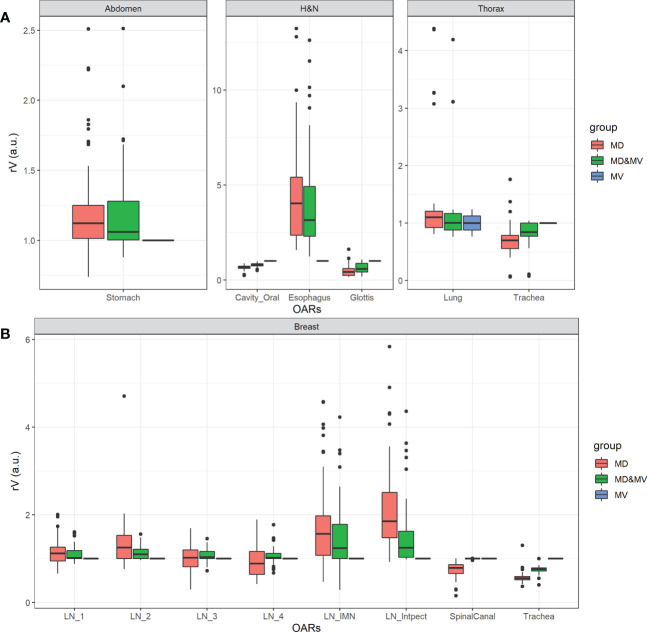
Boxplot of rV of delineated OARs and districts in which the manually adjusted VOIs showed a difference higher than 10% compared to MV alone (i.e., rV lower than 0.9 or higher than 1.1) in less than 75% of MD&MV cases. The relative volume differences observed in the abdomen, H&N, and thorax districts are shown in panel **(A)**, while for breast cancer patients in panel **(B)**. See the definition of **Figure 1** legend.

**Figure 5** shows the boxplot of rVs of organs with a lower agreement (rV lower than 0.9 or higher than 1.1) in less than 75% of MD&MV VOIs. The differences rVs among groups were statistically significantly different using the ANOVA test except for LN3 and LN4. In particular, based on the Tuckey test, there was a statistically significant difference in rV delineated by MD versus MV in all the organs except for LN3, LN4, and lung, while there was a statistically significant difference in rVs delineated by MD&MV versus MV in all the organs except for LN1, LN2, LN3, LN4, spinal canal and lung.

Large discrepancies amongst volumes can be detected in the spinal canal, in hollow organs (or lumen tissue type), and in small organs (e.g., penile bulb) or lymph nodes (see **Figures 4**, **5**).

Among organs with discrepancies, the spinal canal was automatically contoured in all the available CT slices by MVision, while ROs drew it in a limited number of slices, which include the target and a few centimeters above or under the target itself. Another exception regarded the delineation of hollow organs (or lumen tissue type). This type of organ might be more challenging to delineate due to the assumed thickness of the organ wall (trachea, rectosigmoid, rectum, esophagus).

Finally, significant discrepancies amongst volumes were also detected in small organs (e.g., penile bulb) or lymph nodes (see **Figures 4**, **5**). This result is likely related to the CT windows used for manual delineation and the preselected window.

Among cases with more significant discrepancies, the variations in stomach delineation with or without auto-segmentation tool may have been influenced using oral contrast or by the organ’s variability in size and morphology. In any case, the delineation of the stomach is a challenging task.

### 3.3 Metrics

Overall, DSC values increased with the volume of the investigated VOIs in both MD and MD&MV groups. Statistically significant smaller DSC values were obtained for the MD group when compared to MD&MV one in the subgroups with small, medium, and large volumes (i.e.,<50 cc, 50-500 cc and >500 cc, respectively).

In the MD subgroup, DSC values obtained when comparing different slice thicknesses (i.e., ≤ 3 mm and > 3 mm) were not statistically significantly different, with a median value of 0.83 and 0.81, respectively; while in the MD&MV subgroup, they were statistically significantly different with a median value slightly decreasing from 0.99 to 0.98, respectively. Overall, the DSC values obtained in the MD&MV subgroup were statistically significantly higher than MD one. In particular, in three districts (i.e., H&N, male and female pelvis), two CT scanners were used for the image acquisitions. Also, in these subsets of patients, the DSC values obtained in the MD&MV subgroup were statistically significantly higher than MD one, irrespective of slice thickness and CT scanner (see **Supplementary Material Figures S1, S2**). Finally, contrast medium was used in 67%, 80%, and 90% of the patients of the abdominal, H&N, and thoracic districts, respectively. In these cases, the DSC values obtained in the MD&MV subgroup were statistically significantly higher than the MD one, regardless of the presence of the contrast agent (see **Supplementary Material Figure S3**).

**Figure 6:**
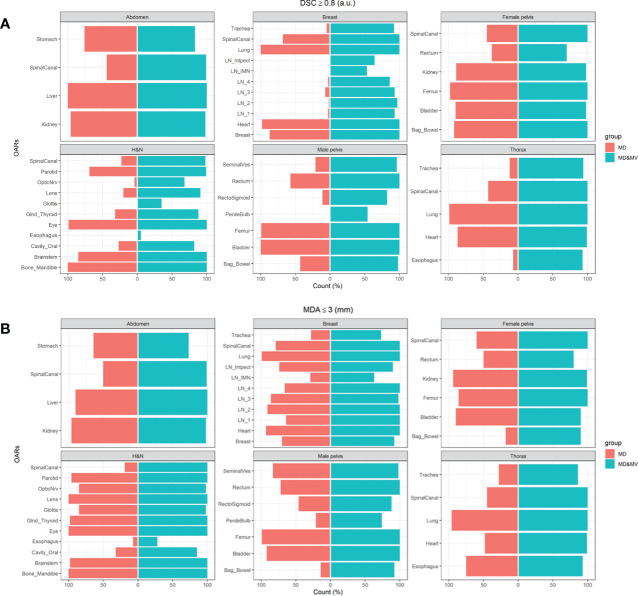
The percentage number of cases in which the **(A)** DSC coefficients of the MD or MD&MV VOIs were higher than 0.8 when compared to MV alone, **(B)** MDA coefficients were lower than 3 mm for the same groups. See the definition of **Figure 1** legend.

**Figure 6** shows the percentage number of cases in which the DSC and MDA indexes, obtained comparing MD and MD&MV VOIs to the MV ones, were higher than 0.8 a.u. and lower than 3 mm, respectively.

The auto-segmentation strongly increases both DSC and MDA values between MD&MV and MV VOIs compared to the same indexes between MD and MV VOIs, as shown in **Figure 6**. Thus, DL-tool could harmonize the delineated OARs between ROs in the same institution and potentially improve multicentric collaborations.

**Figures S4, S5** in **Supplementary Materials** show the DSC and MDA values between MD&MV or MD and MV VOIs in the two subgroups (S and J). These results are substantially similar to the ones of the whole group.

**Figure 7:**
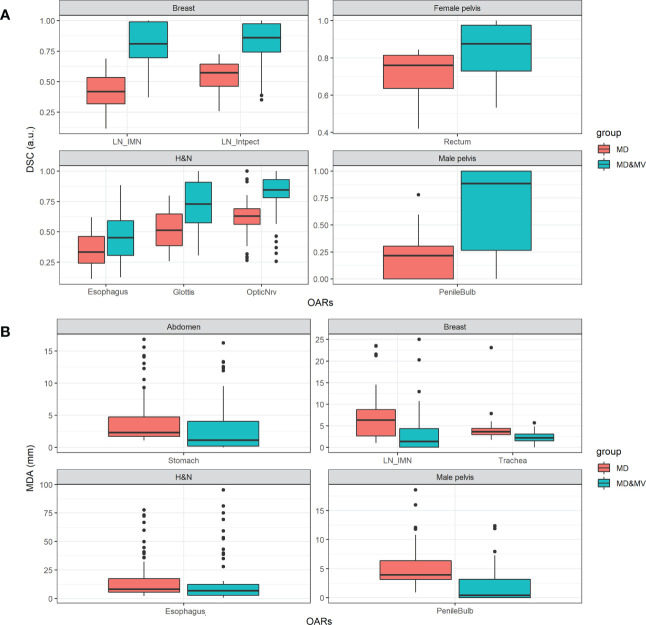
Boxplot of **(A)** DSC and **(B)** MDA values of delineated OARs and districts in which the percentage number of MD&MV higher than 0.8 a.u. and lower than 3 mm, respectively, was lower than 75%. See the definition of **Figure 1** legend.

**Figure 7** shows the boxplot of DSC and MDA of organs in which the percentage number of MD&MV higher than 0.8 a.u. and lower than 3 mm compared to MV alone, respectively, was lower than 75% (as shown in **Figure 6**). The differences among MD and MD&MV groups were statistically significant based on the t-test, except for the MDA values of the esophagus in the H&N district (data not shown).

As for the volumes, the DSC and the MDA coefficients have a large variability among groups and OARs (**Figure 7**) in all the districts, except for the DSC in the abdomen and thorax district and the MDA in the thorax and female pelvis district. The manual adjustment of MV VOIs statistically significantly increases the DSC values (i.e., comparing the DSC between MD&MV and MV VOIs and the DSC between MD and MV VOIs). The median DSC values (comparing MD&MV versus MV VOIs) were higher than 0.8 for all the organs except for the esophagus and glottis in the H&N district.

As expected, the MDA values of MD&MV compared to MV VOIs were statistically significantly lower than the MDA values of MD compared to MV VOIs. The median MDA values (comparing MD&MV versus MV VOIs) were lower than the 3 mm expected for the esophagus in the H&N district.

## 4 Discussion

The increasing development of automated RT workload is expected to lead to a growing number of patients treated with modern accelerators enabling advanced delivery techniques. These accelerators deliver high conformal dose distributions, demanding an accurate delineation of target and OARs to improve tumor control while reducing toxicity. With the rising interest and spreading of machine learning applications in Radiation Oncology, AI auto-segmentation is widely hypothesized to be associated with workflow benefits and time savings, as shown in limited prospective data (23).

Automated organ segmentation from CT images has different applications, including radiation therapy, diagnostic tasks, surgical planning, and patient-specific organ dose estimation. One challenge posed by the DL auto-segmentation approach is requiring a large, manually labeled training dataset. External validation is one of the more relevant tasks to propose a further improvement of DL models in a real-world context (24).

In this study, we evaluated the performance of a DL-based auto-segmentation algorithm on local data, representing an external validation. Specifically, we compared the MD and the MD&MV contours versus the MV ones.

Based on expert contours of ROs with different levels of experience and trained in multiple centers, we measured inter-ROs variability and manual contours and estimated the agreement or disagreement of DL-tool-based contours accordingly to the district and VOIs. In addition, we estimated the inter-ROs variability of DL-tool-based contours manually adjusted.

Unfortunately, large datasets of CT scans for several tumoral sites with anatomical annotations from several radiation oncologists are lacking (25).

Several auto-segmentation methods have been developed and investigated, with DL convolutional neural network methods demonstrating performance improvements (15, 16, 26, 27).

To date, organ auto-segmentation efforts have been mainly focused on adult populations, and several adult CT segmentation datasets have been publicly released. However, Raudaschl et al. (18) indicated the need to have different medical experts performing manual segmentations on the same structure. In other words, assessing the inter-observer variability can help judge the added values of the automatic segmentation.

The results obtained in volume, metrics (DSC, MDA), time saved, and satisfactory score are discussed in the following paragraphs according to the ROs experience, type of scanner, acquisition and reconstruction parameters, presence of contrast medium, and the potential reasons for which the DL-tool correctly did not perform the contour or failed to identify a VOI.

To the best of our knowledge, no one has evaluated the performance of auto-segmentation software (commercial or not commercial) on a cohort of patients comprising multiple body districts (sites). Our dataset provides CT data of six types/sites or RT treatment from two scanners. The images were collected to be used in clinical practice.

### 4.1 Delineated VOIs and time saving

MV generally delineated all the planned VOIs with a few exceptions, likely due to previous surgery, presence of prosthesis or modification of patient anatomy due to external devices. More in details, we distinguished several cases: the first consisted of VOIs not delineated because not recognized (i.e. the bone mandible partially removed in one H&N case due to a hemi-mandibulectomy operation), the second included VOIs appropriately not delineated being surgically removed (i.e., one kidney removed in two abdominal cases due to nephrectomy, seminal vesicles in four male pelvis patients after radical prostatectomy and one lens in a single H&N patient), the third comprised altered patient anatomy due to organs dislocation and deformation for the application of external perineal ultrasound probe (28) (i.e., the penile bulb), while the four involved the replacement of a femoral head with a metal implant or two lens during cataract surgery.

We focused our analysis on all the VOIs available in the investigated CE/FDA-approved version of MVision while acknowledging that key structures, such as the duodenum, small bowel, large bowel, and pancreas, are unavailable for the abdominal district. The only exception was the investigation of loco-regional lymph nodes of the breast, which were available in a research version that we had the opportunity to test. Of note, we investigated the performance of MV delineation of various VOIs included in anatomical sites not yet reported in the literature (e.g., thorax, gynecological,…).

The time saved resulting from our study was up to 92%, 92%, 63%, 73%, 54%, and 72% for the abdomen, thorax, H&N, breast, female and male pelvis, respectively, while the performance declared by the Vendor is up to 95% overall.

Furthermore, a time saving from 0.6 to 17 min using a previous version (version 1.1) of this software was reported by Kiljunen et al. (15) only for the prostate district. These results agree with the ones from our study, showing an absolute segmentation time saving ranging from 3.5 to 18.4 min for the same district.

### 4.2 Volumes

Expert VOIs from senior and junior ROs were manually labeled for up to 29 OARs in six districts. The volumes were similar in larger organs, with a better agreement between manually adjusted VOIs versus MV. In the abdominal area (e.g., pancreas), some organs were less defined due to the limits in the image quality of CT images. According to the clinical practice, we selected images carried out with or without contrast agents; thus, a possible discrepancy among VOIs was expected. In the thorax case, we found closer overlap indices for the breast contour, while a more significant variation was observed in the lymph node delineation.

The spinal canal volume was generally higher in the MV segmentation or the MD&MV one because it was automatically delineated in all the slices of CT, while ROs normally contoured only the volume involved in the treatment.

For this reason, the standard deviations of volumes were generally higher among the MD group than in the MV or MD&MV, while they were similar for larger organs, such as the stomach and liver, completely delineated in all the groups. Overall, the variability of the delineation among MD group was quite high in our analysis, although lower than that reported in the literature in different anatomical districts (15, 29).

The variability of the VOIs was higher in the MD than in the MD&MV group, although manual adjustment of automatic VOIs was required in several organs. For most OARs, the agreement with MV was higher for MD&MV than for MD alone, irrespective of RO experience. This issue is also of relevance for CTV (breast and seminal vesicles) or LN (breast) delineation because the increased consistencies may improve the actual target coverage (25).

### 4.3 Metrics and the satisfactory grade

Many OARs required an adjustment of delineated MV VOIs. Since the agreement between two ROs is considered acceptable with a DSC≥0.8, we observed that the MD&MV increased the agreement among ROs and MV VOIs. Similar consideration was found for the MDA, for which the average distance between VOIs was lower than 3 mm for most OARs.

Similar results for the analysis of DSC and MDA values were also found by considering the S and J subgroups separately, supporting a small impact of the ROs’ experience.

The increase in the DSC index of MD&MV in the breast cancer district agrees with the findings by Byun et al. (30) for delineating several organs. Of note, in our study, the reduction of inter-ROs variability was also found for the same district in lymph node delineation, representing the most challenging VOIs, being smaller volumes and target areas for breast cancer patients with LNs involvement. Moreover, a limitation of the DSC index is that it only considers the overlap between VOIs, irrespective of their shape and orientation. The same limitation applies to MDA.

The average satisfactory grade per district was higher than 4, except for the female pelvis. This score was statistically significantly higher in S than in J ROs. The satisfactory grade was expected to incorporate the time spared and the need for editing, considering all the VOIs within a given district.

ROC curve analysis revealed that higher satisfactory grades (≥4) were statistically significantly associated with the higher mean DSC per patient in S, J, and the whole group, respectively, but not with a higher mean MDA per patient.

### 4.4 Potential applications of our results

Considering the expected impact on patient-specific organ dose estimation, strategies for commissioning and clinical implementation of these algorithms are mandatory before introducing these systems in clinical practice (24). Thus, a QA procedure is recommended before implementation to guarantee that treatments are comparable and consistent with those developed using manual approaches (18). Our approach might be considered a platform for commissioning and QA of DL tools thanks to many patients, districts, and VOIs performed by at least 3 ROs. In addition, we aim to optimize the analysis of the auto-segmentation tool, as a fundamental step in the RT patient workflow, by allocating better resources and sparing time for the semi-automatic analysis. For this reason, we developed a new/innovative methodology/strategy for the QA/validation of the segmentations, auto-segmentations, and auto-aided-segmentations in our operative unit to support RT staff in clinical practice.

Our dataset enables the evaluation and development of organ auto-segmentation algorithms in a large population of patients undergoing RT who exhibit organ shape and size variations across gender and age. We plan to use this dataset and methods to evaluate other algorithms’ performance under standardized circumstances comparable to clinical practice.

Of note, one issue remains on the dataset used, mainly represented by the inter-RO variability of expert contours used for the DL-tool training and validation. Olsson et al. (19) showed for a single VOI (i.e., rectum) that, the DSCs of retrained MVision model were 0.89 ± 0.07 while the one of the clinical and the original MVision tool were 0.87 ± 0.07 and 0.86 ± 0.06, respectively, thus suggesting that the DSC variability remains similar after the model retraining. Based on these results, our study focused on assessing the performance of the MVision algorithm using an external validation dataset, considering the inter-RO variability of several expert ROs, all applying the ESTRO and international delineation guidelines.

As a future development, in case of unsatisfactory contouring, the present tool could be trained, for example, using patients undergoing surgery or with altered anatomy, to provide robust and high-quality data.

## 5 Conclusion

Our analysis revealed the positive impact of introducing and validating a novel CE- and FDA- approved commercial DL tool for automatic segmentation in terms of; i) a high level of clinicians’ satisfaction, particularly for complex cases including large and numerous organs, ii) saving time, and iii) improving the consistencies of VOIs amongst different ROs.

Finally, our study permitted the creation of a platform including CT images and multiple expert VOI contours for six districts to commission new auto-segmentation tools and QA protocols.

## Data availability statement

All data generated or analysed during this study are included in this published article (and its **Supplementary Information Files**).

## Ethics statement

The studies involving human participants were reviewed and approved by Ethics Committee of IRCCS Azienda Ospedaliero-Universitaria di Bologna (Identification codes of the projects: ICAROS 311/2019/Oss/AOUBo, ESTHER 973/2020/Oss/AOUBo, PORTO 533/2021/Oss/AOUBo, PAULA 201/2015/O/Oss, BREATH 229/2019/Oss/AOUBo). The patients/participants provided their written informed consent to participate in this study.

## Author contributions

Conceptualization: LS. Methodology and formal analysis: LS, SS and MS. Investigation: LS, MS, and GP. Data curation: GP, MS, SS, IA, AA, AB, SB, RC, LC, ED, CMD, EG, AGu, AGa, MF, VL, FM, MN, NR, GS, GT, DV, and AZ. Writing—original draft preparation: SS, MS, and LS. Writing—review and editing: GP, AA, SB, and AGM. Visualization: SS. Supervision: LS and AGM. Funding acquisition: LS. All authors contributed to the article and approved the submitted version.
