# Supplementary material for: How smart is artificial intelligence in organs delineation? Testing a CE and FDA-approved Deep-Learning tool using multiple expert contours delineated on planning CT images
Source: Front Oncol. 2023 Mar 2;13:1089807. doi: 10.3389/fonc.2023.1089807 (PMC10019504; doi:10.3389/fonc.2023.1089807)
Supplement: Supplementary file 1 [file Presentation_1.pptx]

## Slide 1
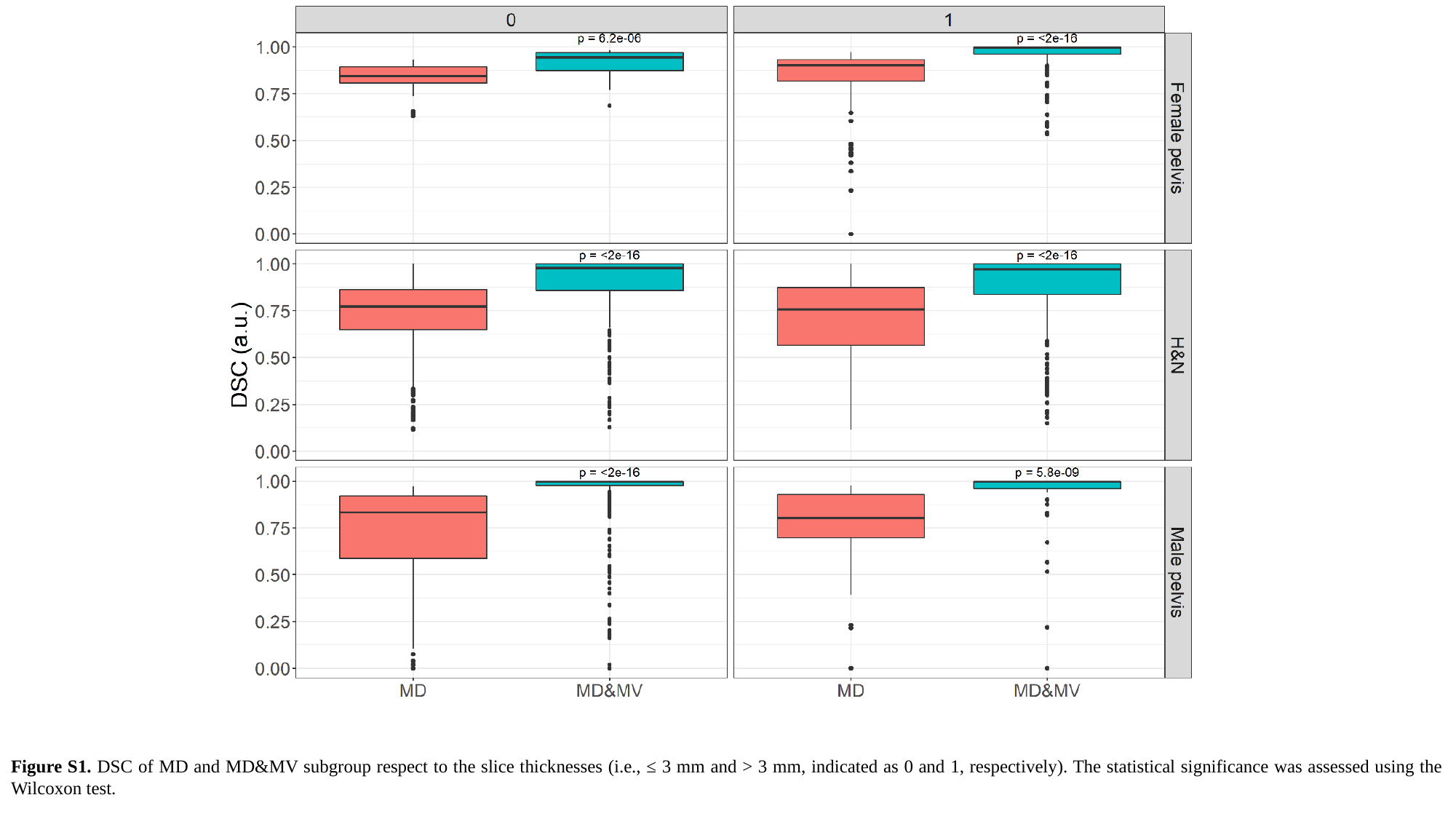

Figure S1. DSC of MD and MD&MV subgroup respect to the slice thicknesses (i.e., ≤ 3 mm and > 3 mm, indicated as 0 and 1, respectively). The statistical significance was assessed using the Wilcoxon test.

## Slide 2
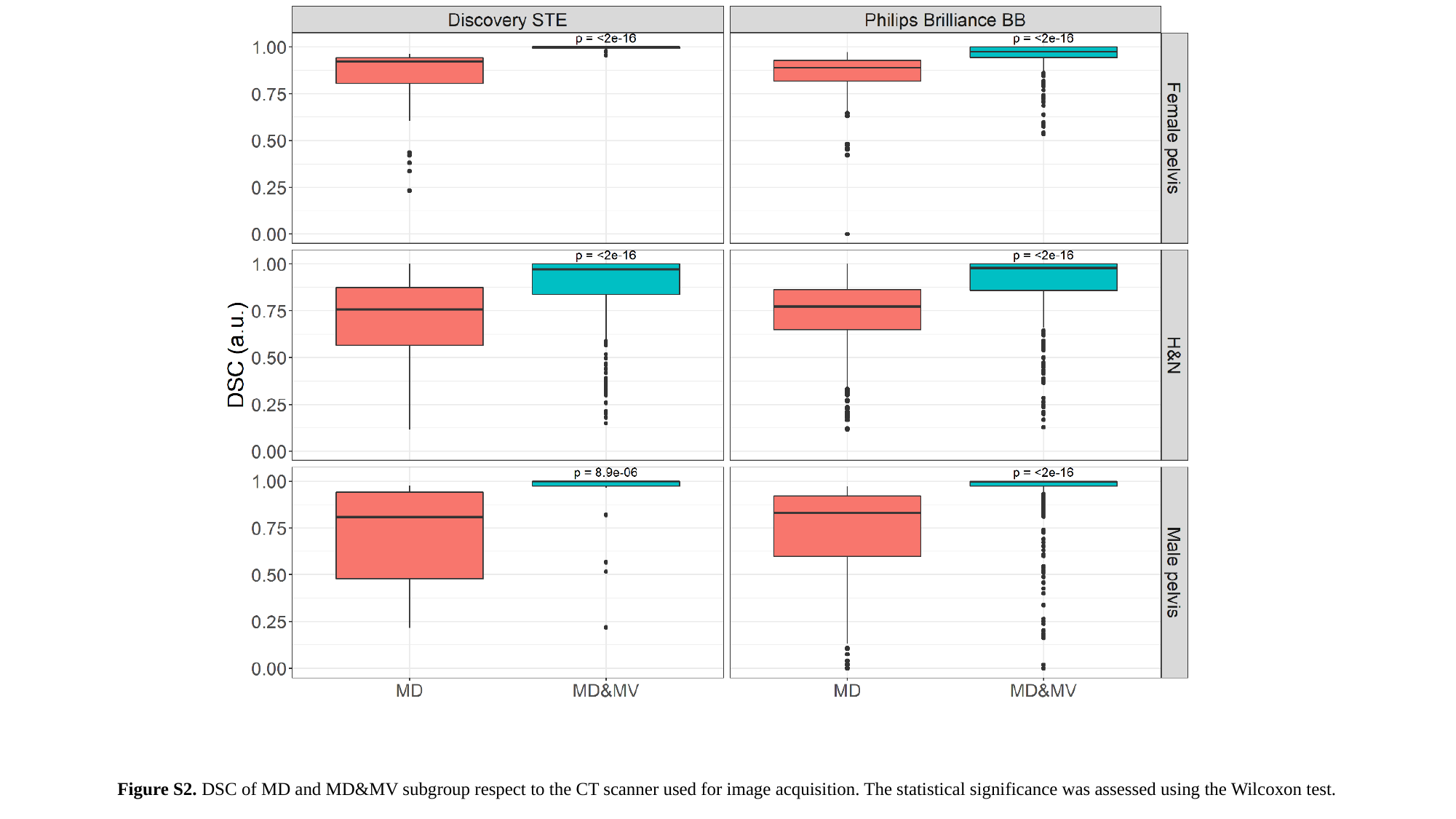

Figure S2. DSC of MD and MD&MV subgroup respect to the CT scanner used for image acquisition. The statistical significance was assessed using the Wilcoxon test.

## Slide 3
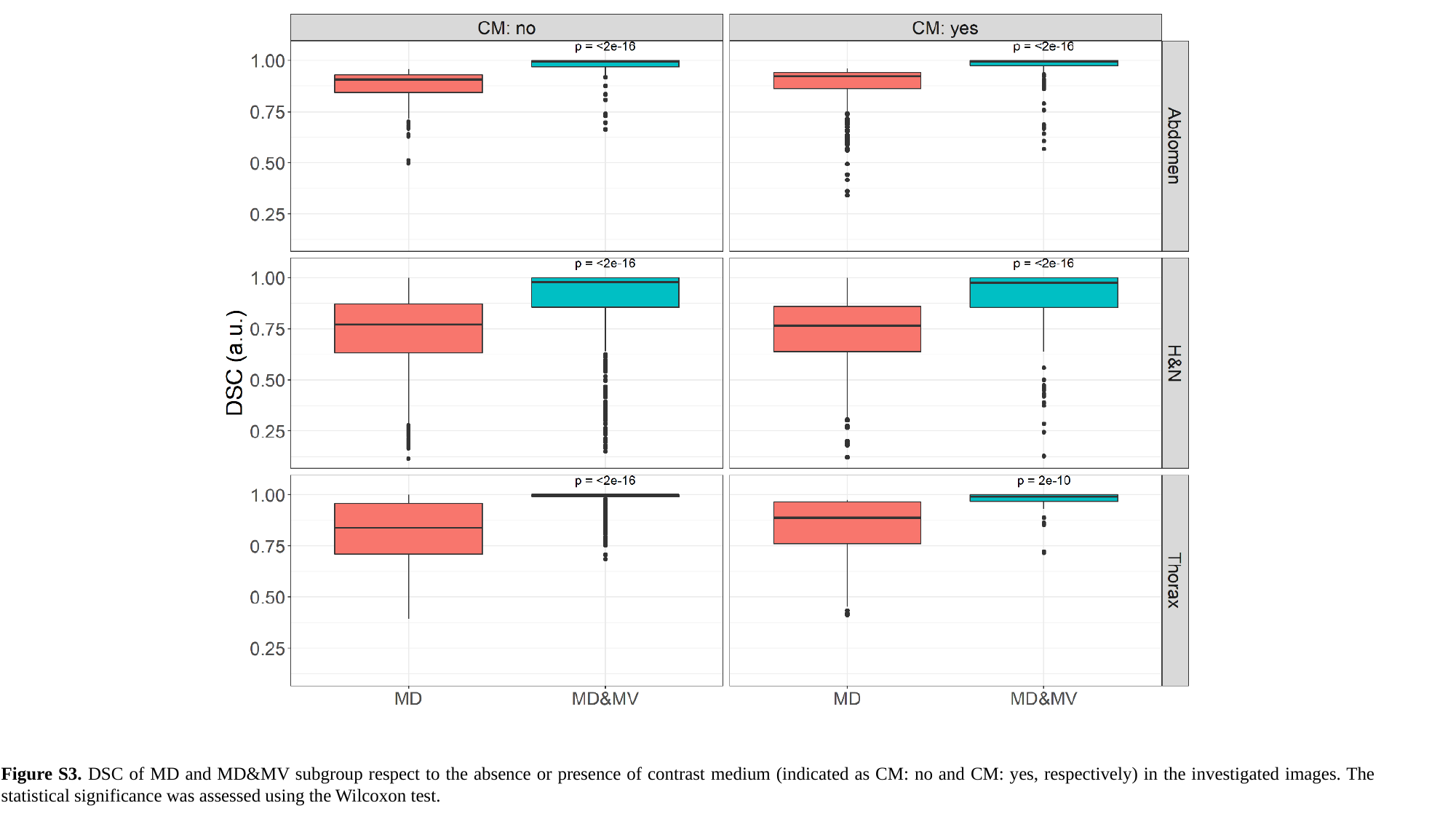

Figure S3. DSC of MD and MD&MV subgroup respect to the absence or presence of contrast medium (indicated as CM: no and CM: yes, respectively) in the investigated images. The statistical significance was assessed using the Wilcoxon test.

## Slide 4
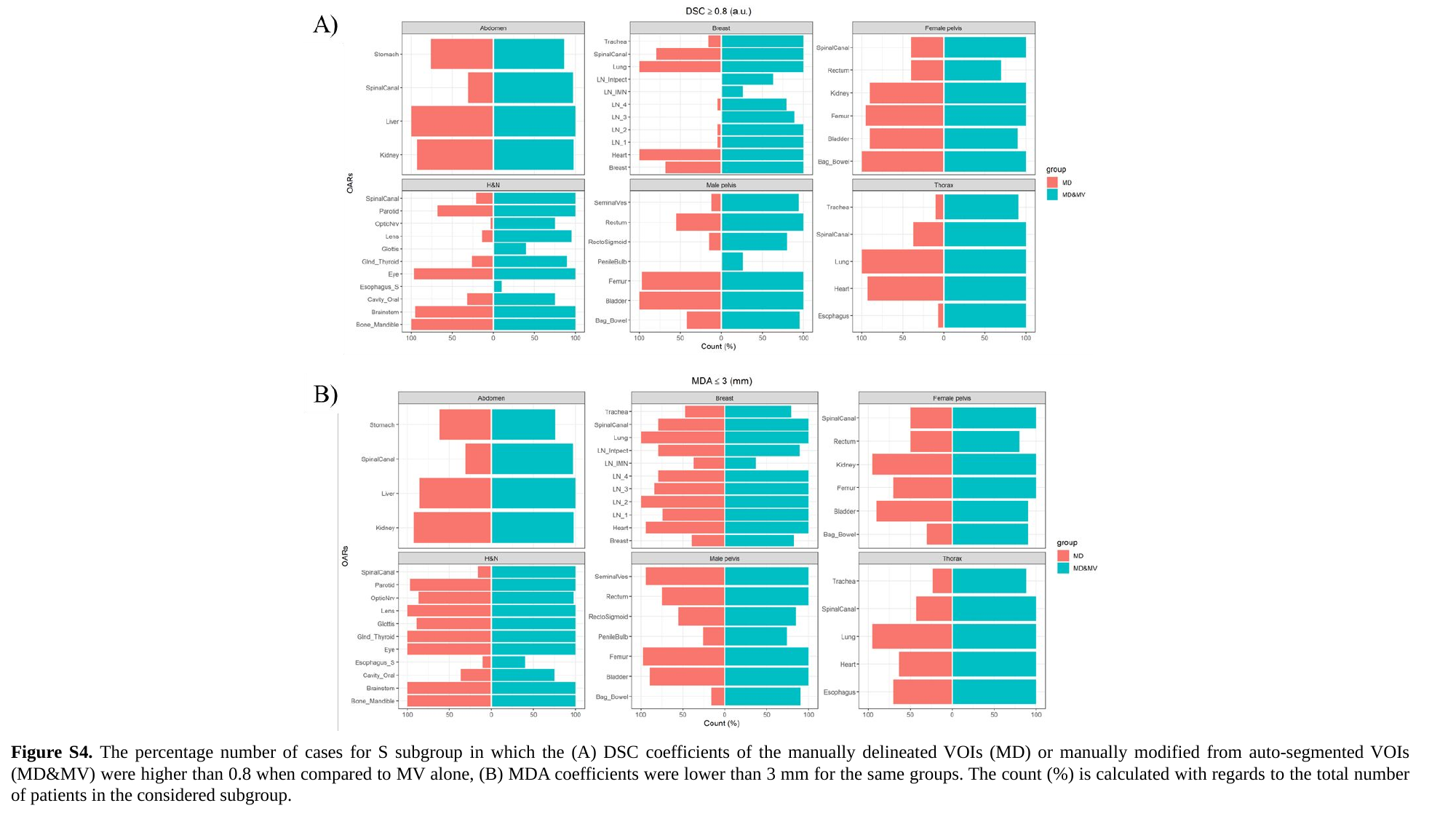

Figure S4. The percentage number of cases for S subgroup in which the (A) DSC coefficients of the manually delineated VOIs (MD) or manually modified from auto-segmented VOIs (MD&MV) were higher than 0.8 when compared to MV alone, (B) MDA coefficients were lower than 3 mm for the same groups. The count (%) is calculated with regards to the total number of patients in the considered subgroup.

## Slide 5
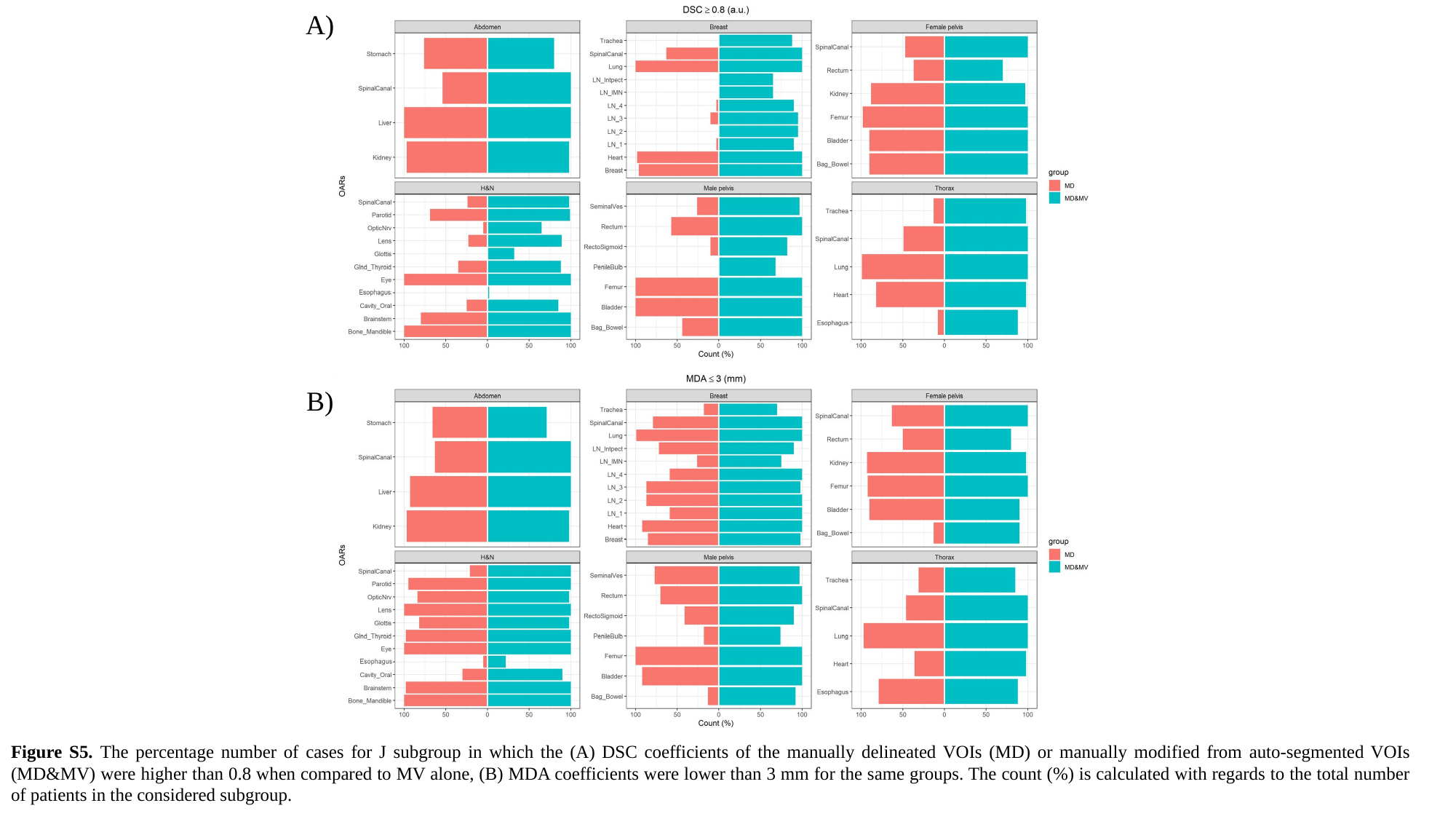

A)
B)
Figure S5. The percentage number of cases for J subgroup in which the (A) DSC coefficients of the manually delineated VOIs (MD) or manually modified from auto-segmented VOIs (MD&MV) were higher than 0.8 when compared to MV alone, (B) MDA coefficients were lower than 3 mm for the same groups. The count (%) is calculated with regards to the total number of patients in the considered subgroup.
